# Orexin receptors within the nucleus accumbens shell mediate the stress but not drug priming-induced reinstatement of morphine conditioned place preference

**DOI:** 10.3389/fnbeh.2013.00144

**Published:** 2013-10-10

**Authors:** Keke Qi, Chuguang Wei, Yonghui Li, Nan Sui

**Affiliations:** ^1^Key Laboratory of Mental Health, Institute of Psychology, Chinese Academy of SciencesBeijing, China; ^2^Institute of Psychology, University of Chinese Academy of SciencesBeijing, China; ^3^The Core Facility, Institute of Psychology, Chinese Academy of SciencesBeijing, China

**Keywords:** orexin, the nucleus accumbens shell, morphine, CPP, stress-induced reinstatement, drug priming-induced reinstatement

## Abstract

Orexins are found to participate in mediating stress-induced drug relapse. However, the neuroanatomical basis that orexin transmission modulates stress-induced drug seeking remains unknown. The nucleus accumbens shell (NAcSh), best known for its role in appetitive and negative motivation via dopamine receptors, is likely to be the potential important brain area where the orexin system mediates stress-induced drug relapse since the function of dopamine system in the NAcSh can be regulated by orexin transmission. In the present study, a morphine conditioned place preference (CPP) model was used to determine whether the two types of orexin receptors would be involved into footshock-induced and/or drug priming-induced CPP reinstatement differentially. The results showed that blockade of orexin-1 or orexin-2 receptor in the NAcSh significantly attenuated stress-induced morphine CPP reinstatement, but neither of the orexin antagonists had any effect on morphine priming-induced reinstatement. These findings indicate that the NAcSh is a brain area through which orexins participate in stress but not drug priming-induced relapse of opioid seeking.

## Introduction

Orexins, including orexin A (OXA) and orexin B (OXB), are neuropeptides derived exclusively from a small group of neurons in the lateral and perifornical areas of the posterior hypothalamus. They act at two receptor subtypes, orexin-1 receptor (OX1R) and orexin-2 receptor (OX2R; de Lecea et al., 1998; Sakurai et al., 1998). Despite having a restricted distribution, orexin neurons project widely to many brain regions where orexins have been implicated in feeding, sleep, arousal, and so on (Chemelli et al., 1999; Sutcliffe and de Lecea, 2002; Taheri et al., 2002). Recently, mounting evidence has shown that orexins are involved in different components of drug addiction (Plaza-Zabala et al., 2012), including drug reward (Harris et al., 2005), drug reinforcement (Espana et al., 2010), drug withdrawal (Georgescu et al., 2003) and drug seeking behaviors (Boutrel et al., 2005). The specific role of orexin receptors in drug reward and reinforcement remains controversial (Borgland et al., 2006; Smith et al., 2009; Shoblock et al., 2011; Voorhees and Cunningham, 2011; Plaza-Zabala et al., 2012), but it is quite clear that the orexin system was involved into different types of drug seeking induced by stress, cue and context associated with drug reinforcement (Boutrel et al., 2005; Dayas et al., 2008; Smith et al., 2010). It indicates that orexin transmission may uniquely modulate drug relapse-like behaviors but may not play a key role in the reinforcement of drugs of abuse that maintains the ongoing drug taking behaviors.

The specific brain site which orexin transmission regulates drug relapse has drawn dramatic attention. Ventral tegmental area (VTA) is likely to be the most importantly potential brain area since it receives heavy orexin fibers (Peyron et al., 1998) and has been shown to express densely two types of orexin receptors in dopamine neurons (Narita et al., 2006). Moreover, electrophysiological evidence shows that orexin applied to midbrain slices increases the VTA dopamine neurons excitability (Korotkova et al., 2003), and intra-VTA infusion of orexin stimulates dopamine release (Narita et al., 2006; Vittoz and Berridge, 2006; Espana et al., 2010). Actually, behavioral studies have shown that intra-VTA injection of orexins reinstate the extinguished morphine and cocaine preference (Harris et al., 2005; Wang et al., 2009), and blockade of OX1R in the VTA attenuate cue-induced cocaine reinstatement (James et al., 2011) but not stress-induced cocaine reinstatement (Wang et al., 2009). This convincing evidence indicates that VTA is the crucial brain structure at which orexin transmission mediates cue-induced drug seeking behaviors via transforming the environmental stimuli into salient incentives (Stuber et al., 2008; Plaza-Zabala et al., 2012), whereas the brain site at which orexin system modulate stress and drug priming-induced drug seeking behaviors remains to be determined.

Recent studies have shown an important role of orexin transmission in the stress regulation (Berridge et al., 2010; Lungwitz et al., 2012); stress-related stimuli enhance the activity of orexin neurons in the dorsal medial part of the hypothalamus (Harris et al., 2005; Johnson et al., 2012), and central injections of orexins increase the release of adrenocorticotrophic hormone (ACTH) and corticosterone (Kuru et al., 2000; Samson et al., 2002) and induce stress-like behaviors in rats (Li et al., 2010). Antagonism of orexin receptors attenuates fear and anxiety-like behaviors (Johnson et al., 2010; Li et al., 2010). Therefore, it is not surprising that systemic blockade of OX1R prevented the stress-induced cocaine reinstatement, and the cocaine reinstatement induced by orexin injection is prevented by antagonism of noradrenergic and corticotropin-releasing factor (CRF) systems (Boutrel et al., 2005). However, to our knowledge, few studies have examined the brain mechanisms by which orexin transmission regulates stress-induced drug seeking behavior.

The nucleus accumbens (NAc), best known for its role in mediating appetitive motivation related to drug relapse and aversive motivation related to stress-like behaviors (Mcfarland and Kalivas, 2001; Reynolds and Berridge, 2002; Mcfarland et al., 2004), is likely to be the potential candidate at which the orexin system may modulate stress-induced drug relapse. The NAc consists of two subregions, referred to as the shell of the NAc (NAcSh) and the core of the NAc (NAcC; Meredith, 1999). Anatomical studies show that orexinergic fibers are mainly distributed in the dorsomedial caudal part of the NAcSh (Baldo et al., 2003), the unique area within the NAc that regulates stress-related behaviors via dopamine receptors (Faure et al., 2008; Richard and Berridge, 2011). Interestingly, it is also found that the orexin fibers intermingle heavily with dopamine fibers in the caudal NAcSh (Baldo et al., 2003), indicating orexin transmission can modulate dopamine release in this area. Indeed, an electrophysiological study reveals that an interaction between the orexinergic and dopaminergic systems occurs in the NAcSh, presumably via OX2R (Mori et al., 2011), and intra-NAcSh injection of orexin increase dopamine concentration in this area (Patyal et al., 2012). More importantly, local injection of orexins in the NAcSh potentiated and OX1R antagonist attenuated dopamine receptor agonist-induced turning behaviors (Kotani et al., 2008). Dopamine D1 receptor in the NAcSh has been shown to participate in stress-induced reinstatement (Shaham and Stewart, 1996; Shaham et al., 2003; Mcfarland et al., 2004). Thus, it is reasonable to postulate that the NAcSh is likely to be a potential brain area where orexin transmission modulates the function of dopamine system to participate in stress-induced reinstatement, and may also regulate drug-primed reinstatement since the role of the NAcSh dopamine receptor in drug priming-induced drug seeking behaivors has been well established (Anderson et al., 2003; Bachtell et al., 2005; Schmidt et al., 2006).

Both types of orexin receptors are reported to be expressed in the NAcSh, where OX1R level is relatively lower than that of OX2R (Marcus et al., 2001; Cluderay et al., 2002; Martin et al., 2002). OX1R, which is selective for OXA, and OX2R, which is nonselective for both OXA and OXB, are coupled to different G proteins, which indicates that OX1R and OX2R may have different functions (Sakurai et al., 1998; Zhu et al., 2003; Faedo et al., 2012). Growing evidence has shown that OX1R signaling is more involved in reward seeking, whereas OX2R signaling is involved in arousal and stress-related behaviors (Lin et al., 1999; Willie et al., 2003; Akanmu and Honda, 2005; Harris and Aston-Jones, 2006; Dugovic et al., 2009; Smith et al., 2009; Wang et al., 2009; Plaza-Zabala et al., 2012). It has been found that chronic drug exposure causes long-lasting up-regulation of OX2R in the NAc (Zhang et al., 2007), and orexin transmission mediates the function of dopamine system in the NAcSh mostly via OX2R (Mori et al., 2011). We speculate that stress and drug priming-induced drug seeking behaviors may be modulated differentially by OX1R and OX2R. Probably, OX2R would play a more important role in the stress-induced reinstatement. In the present study, OX1R and OX2R antagonists are locally injected into the NAcSh to examine whether they modulate stress and/or drug priming-induced drug relapse with an reinstatement paradigm, which is widely used in the studies of drug relapse (Shaham et al., 2003).

## Materials and methods

### Subjects

A total of 135 male Sprague-Dawley rats (Charles River, Beijing, China) were used in these experiments. The rats were housed individually in plastic cages on a 12 h light/12 h dark cycle (lights on at 07:00) in a temperature and humidity-controlled colony room with food and water available at all times. All the behavioral tests were conducted during the light phase. The experimental procedures followed the National Institutes of Health Guide for Care and Use of Laboratory Animals (Publication No. 85–23, revised 1985) and the experimental protocol was approved by Research Ethics Review Board of Institute of Psychology, Chinese Academy of Sciences.

### Surgery and morphine treatment

After a week of acclimatization, rats were anesthetized with equithesin (0.3 ml/100 g, i.p.) to implant stainless steel guide cannulas (23 gauge, Plastics One, Roanoke, VA, USA) bilaterally into the NAcSh (1.7 mm anterior to bregma, 0.8 mm bilateral to the midline, and 5.5 mm ventral to the skull, with the incisor bar at 3.3 mm below intraaural line). Three stainless steel screws were attached on the skull to anchor the guide cannula, and dental cement was used to secure the guide cannulae in place. The capped stylets (Plastics One, Roanoke, VA, USA) were inserted to prevent occlusion. Then the rats were placed in their home cages to recover for a week. Eight days after the surgery, the rats were injected with escalating morphine doses 5.0 mg/kg, 10.0 mg/kg, 20.0 mg/kg, 30.0 mg/kg, 40.0 mg/kg and 50.0 mg/kg (i.p.) for 6 days (each day with a different dose and each dose was given twice a day) to induce drug dependence status (Li et al., 2011). After the repeated morphine injection, the rats went through 5 days abstinence periods in the home cages and were handled for 5 min every day during the last 3 days of the abstinence periods to acclimatize them to the experimenters’ manipulation.

### Drugs and microinjection

Morphine hydrochloride (Qinghai pharmaceutical, China), SB334867, an OX1R antagonist (Tocris, U.K.), TCSOX229, an OX2R antagonist (Tocris, U.K), DMSO (Sigma, U.S.) and saline (NaCl 0.9%) were used in the experiments. Morphine (5.0 mg/ml, 3.0 mg/ml) and TCSOX229 (6.0 μg/μl, 20.0 μg/μl) were dissolved in saline; SB334867 was dissolved in DMSO to the concentration of 6.0 μg/μl, 20.0 μg/μl immediately before use. Three days before the drug injection procedure, all the rats were transferred to the behavioral test room and stayed there for 2 h and received mock microinjection daily to minimize stress. During the microinjection period, each rat was gently held while the stylet was removed. The drug or vehicle (0.5 μl) was injected through an injector cannula (30 gauge, Plastics One, Roanoke, VA, USA) which protruded 2.0 mm below the guide cannula tip. An infusion pump mounted with a glass microsyringe was used to deliver the drug at the rate of 0.2 μl/min over 2 min, and the injector stayed in the guide cannula one more minute to prevent fluid backflow.

### Conditioned place preference (CPP) procedures

#### Apparatus

Seven identical black plastic boxes, measuring 80 × 40 × 50 cm (L × W × H), were used for the Conditioned place preference (CPP) test. The boxes were separated by a guillotine door into two compartments with distinct visual and tactile cues. One side had a dot floor with white horizontal stripes on the walls, and the other side had a smooth floor with vertical stripes on the wall.

#### Behavioral procedures

The procedures consisted of four phases: adaptation, pretest, conditioning and posttest. On day 1 (adaptation), the rats were given access to the boxes for 15 min to reduce the novelty and stress. During the pretest session (Day 2 and Day 3), the rats were placed into the middle of the box and allowed to explore freely. The time spent and distance travelled on both sides were recorded for 15 min by a camera and analyzed by a computer with professional software (Taiji Software Company, Beijing, China). The average time spent in one compartment of the box both days was used as the initial preference score; the pretest data showed that most rats preferred the dot floor side. So a biased CPP procedure was used in the conditioning phase in which the rats received a morphine injection (5.0 mg/kg, i.p.) prior to placement in their non-preferred chamber for 45 min in the morning, and the same volume of saline in the reverse chamber in the afternoon each day for 3 days. A 15 min posttest expression session was done after the conditioning, during which the rats could go through the two chambers freely with the door open. The criterion for the development of CPP is that the time spent in the morphine-paired chamber was 100 s more than that in the pretest session. Otherwise, they would receive two more morphine conditioning sessions until the rats could reach the criterion. After acquisition of morphine CPP, rats were given 9–12 extinction training sessions with the same procedure like conditioning phase except that saline was paired with each chamber. The 15 min posttest session was done to assess the extinction effect. The extinction training continued until the time spent in morphine paired side was at least 80 s less than that during expression test.

#### Experimental design

##### Experiment 1: Effects of microinjection of the OX1R and OX2R antagonists into the NAcSh on the footshock-induced reinstatement of morphine CPP

Six groups of rats (67 rats) were used in the experiment. The control group (*n* = 11) received intra-NAcSh vehicle injection without footshock, and another five groups of rats received different doses of OX1R or OX2R antagonists microinjection into the NAcSh (0.5 μl saline (*n* = 12), SB334867 (3.0 μg/0.5 μl, *n* = 11 and 10.0 μg/0.5 μl, *n* = 11) and TCSOX229 (3.0 μg/0.5 μl, *n* = 11 and 10.0 μg/0.5 μl, *n* = 11)) 15 min before the footshock stress. The electrical footshock was administered as previous studies (Wang et al., 2002, 2006). Briefly, the rats were put into the shock chamber (22 × 22 × 16 cm, Med associates, Inc., St. Albans, VT, USA) and received 15 min footshock (0.8 mA, 0.5 s) with an average 60 s interval. After the footshock procedure, the rats were placed into the CPP box for 15 min to assess the effect of footshock stress on the reinstatement of morphine CPP.

##### Experiment 2: Effects of microinjection of the OX1R and OX2R antagonists into the NAcSh on the morphine-induced reinstatement of morphine CPP

Another six groups of rats (68 rats) were used in this experiment. The control group rats (*n* = 11) received vehicle microinjection into the NAcSh and intraperitoneal saline injection, the other five groups of rats received different doses of OX1R or OX2R antagonists mincroinjection into NAcSh (0.5 μl saline (*n* = 11), SB334867 (3.0 ug/0.5 μl, *n* = 11 and 10.0 μg/0.5 μl, *n* = 11) and TCSOX229 (3.0 μg/0.5 μl, *n* = 12 and 10.0 μg/0.5 μl, *n* = 12)) 15 min before the morphine challenge (3.0 mg/kg, i.p.). The rats were placed into the CPP boxes for 15 min and allowed to explore freely to evaluate the effect of morphine challenge injection on the reinstatement of CPP.

### Cannula verification

At the end of these experiments, the rats were deeply anesthetized with chloral hydrate (40.0 mg/kg) and then perfused transcardially with heparinized saline followed by 500 ml ice-cold 4.0% paraformaldehyde in 0.1 M phosphate buffer (PB, pH 7.4). A freezing microtome was used to get coronal sections from the injection site at 40 μm. The brain slices were processed for cresyl violet staining as previously done in our laboratory (Han et al., 2010).

### Statistical analysis

The preference score (time spent in the morphine-paired side) was analyzed by paired samples *t* test to assess the acquisition and extinction of morphine CPP. The shift score (preference score in reinstatement test minus preference score in extinction test) among different drug treatment groups was analyzed by one-way ANOVA. Post-hoc analysis with LSD test was employed to determine if difference between groups was significant. All data was shown as mean ± SEM and analyzed with SPSS 16.0 software for Windows.

## Results

### Cannula verification

**Figure 1:**
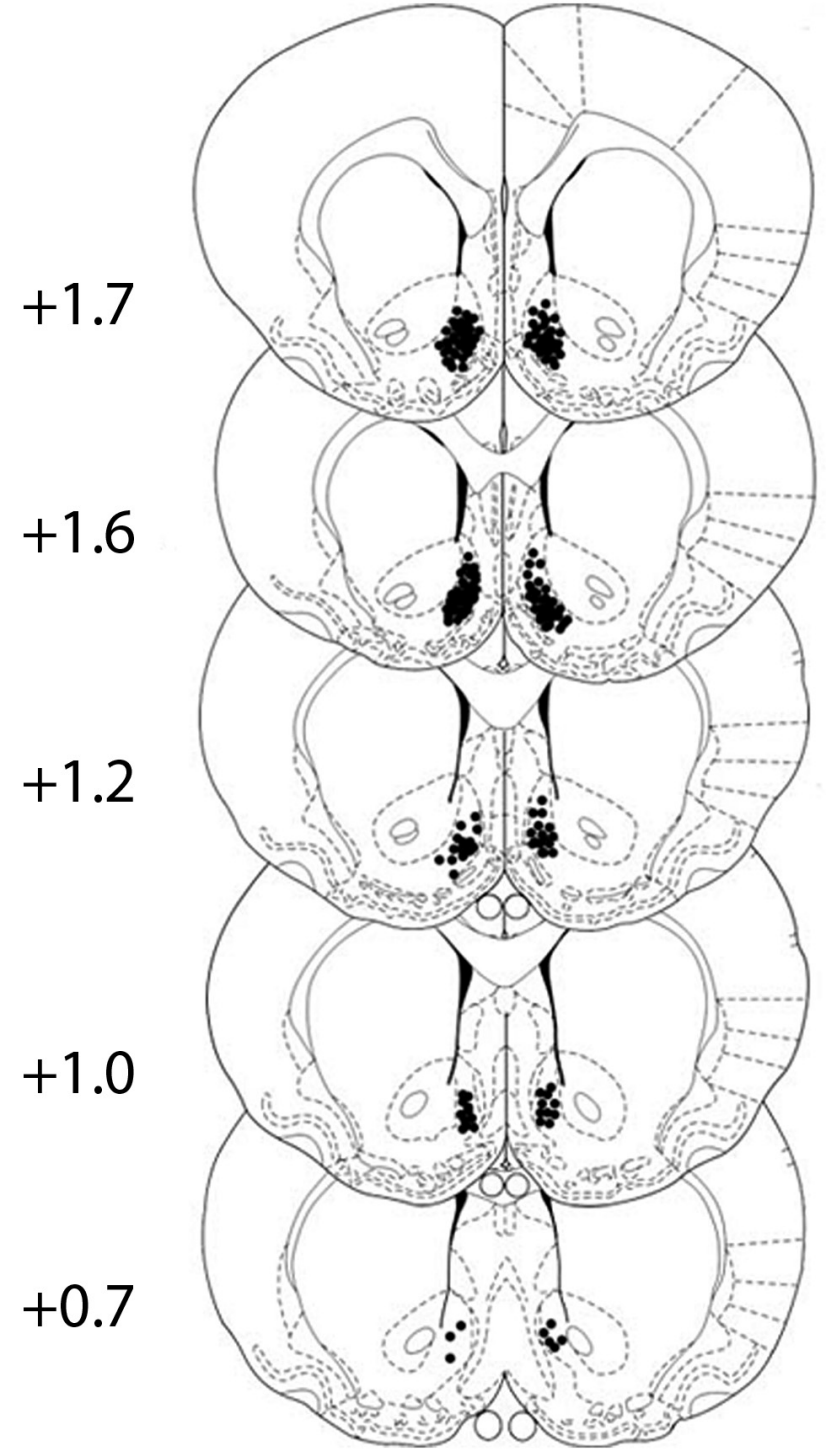
**All cannula placements in corresponding levels of the NAcSh.** Numbers to the left indicate the approximate rostrocaudal plane posterior to bregma. Black dots show the location of the tip of the injector cannula for the rats. The figure is adapted from diagrams of a stereotaxic atlas of the rat brain (George Paxinos, 1996).

**Figure 2:**
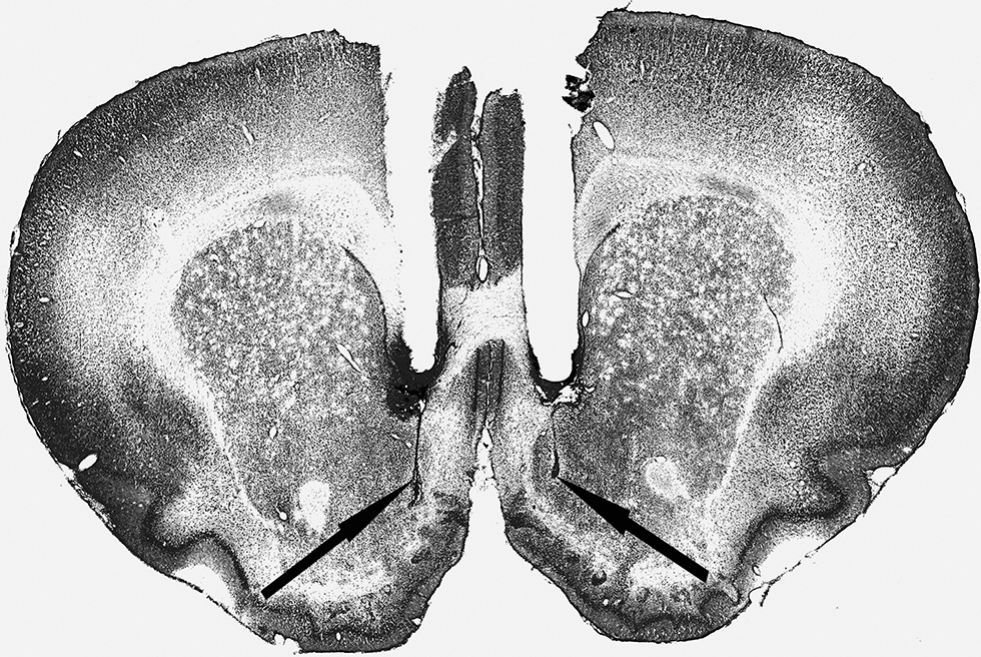
**Representative photomicrograph of the coronal section**.

Cannula placement was checked by postmortem histological verification. The rats with cannula placements within the following coordinate scopes were used in the statistical analysis: 0.7 mm–1.7 mm anterior to bregma, 0.3 mm–1.6 mm bilateral to the midline, and 6.2 mm–8.2 mm ventral to the skull (Figure 1). Thirteen rats were removed because their placements were outside the scopes. A representative photomicrograph of the microinjection sites was presented in Figure 2.

### Acquisition and extinction of morphine induced CPP

Paired samples *t* test showed that rats developed an obvious CPP after 3–5 days of conditioning training, compared to their pretest data (*t*(121) = 29.617, *p* < 0.0001, figure not shown). After 9–12 extinction training, the preference score significantly decreased compared to the score measured in the expression test (*t*(121) = 16.412, *p* < 0.0001, figure not shown). It suggested the acquired CPP was obviously extinguished.

### Experiment 1: OX1R or OX2R antagonist inhibited the footshock-induced reinstatement of morphine CPP

**Figure 3:**
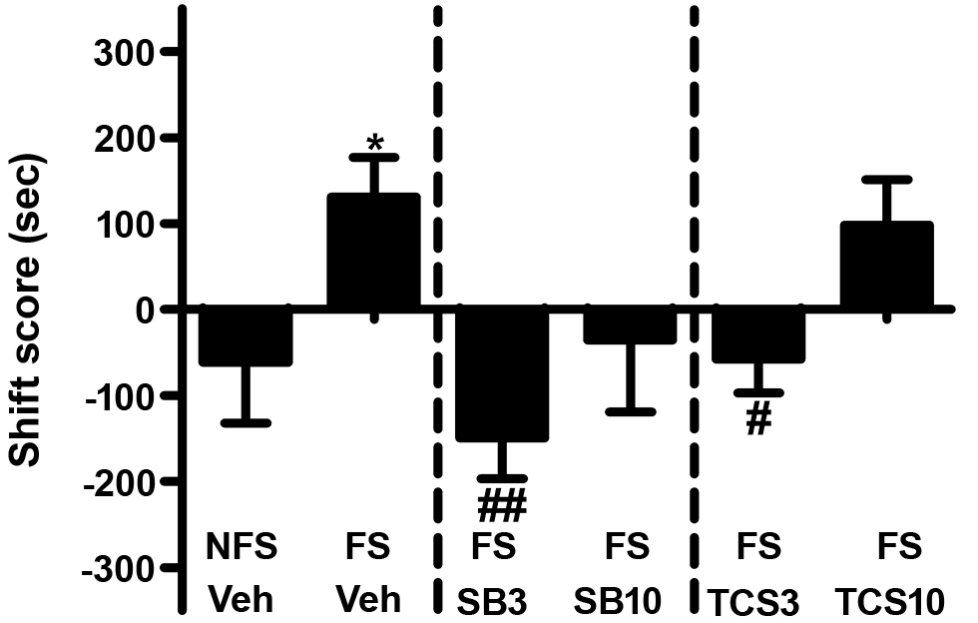
**Effects of microinjections of vehicle (Veh), SB334867 (SB) at 3.0 or 10.0 μg (SB3 and SB10), or TCSOX229 (TCS) at 3.0 or 10.0 μg (TCS3 and TCS10) in the NAcSh on footshock-induced reinstatement of morphine CPP.** *p < 0.05, the footshock group (FS + Veh) compared to the control group non-footshock (NFS + Veh). ^#^*p* < 0.05, ^##^*p* < 0.01, compared to the FS + Veh. All data are expressed as mean ± SEM.

**Figure 4:**
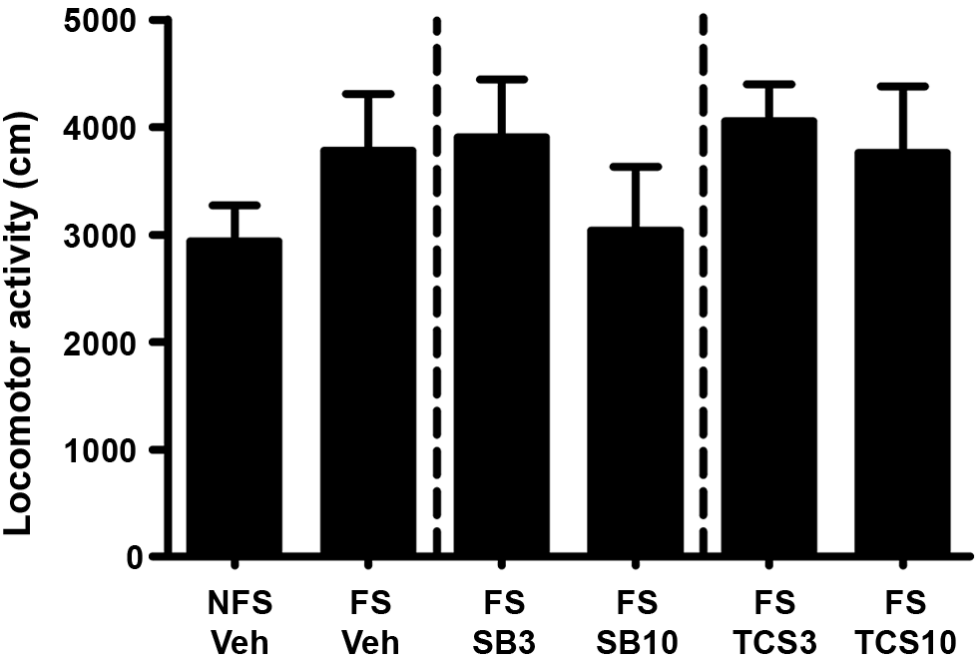
**Mean locomotor activity of all the groups in experiment 1.** All data are expressed as mean ± SEM.

As depicted in Figures 3 and 4, independent samples *t* test revealed that the rats receiving footshock stress spent more time in the morphine-paired side compared to that in the nonshock rats (*t*(13.716) = 2.264, *p* = 0.040), indicating a significant reinstatement of morphine CPP. To examine the effect of OX1R or OX2R antagonist on the footshock induced reinstatement, the rats received two doses of SB334867 or TCSOX229 microinjection into the NAcSh. The one-way ANOVA revealed a significant difference among the groups (*F*(2,24) = 4.475, *p* = 0.022), and the post-hoc test suggested that the low dose of SB334867 (3 μg) blocked the footshock-induced reinstatement (*p* = 0.007). A significant difference was also found among the TCSOX229 groups and vehicle group (*F*(2,26) = 4.210, *p* = 0.026) by the one-way ANOVA, and the LSD test showed that the low dose of TCSOX229 (3 μg) also blocked the footshock-induced reinstatement (*p* = 0.012). Neither orexin receptor antagonists had any effect on the locomotor activity (*F*(2,24) = 0.726, *p* = 0.494; *F*(2,26) = 0.091, *p* = 0.913). It suggested that the inhibition effect of OX2R antagonist on the reinstatement was not caused by the motor impairment.

### Experiment 2: Neither OX1R nor OX2R antagonists had any effect on morphine-induced reinstatement of morphine CPP

**Figure 5:**
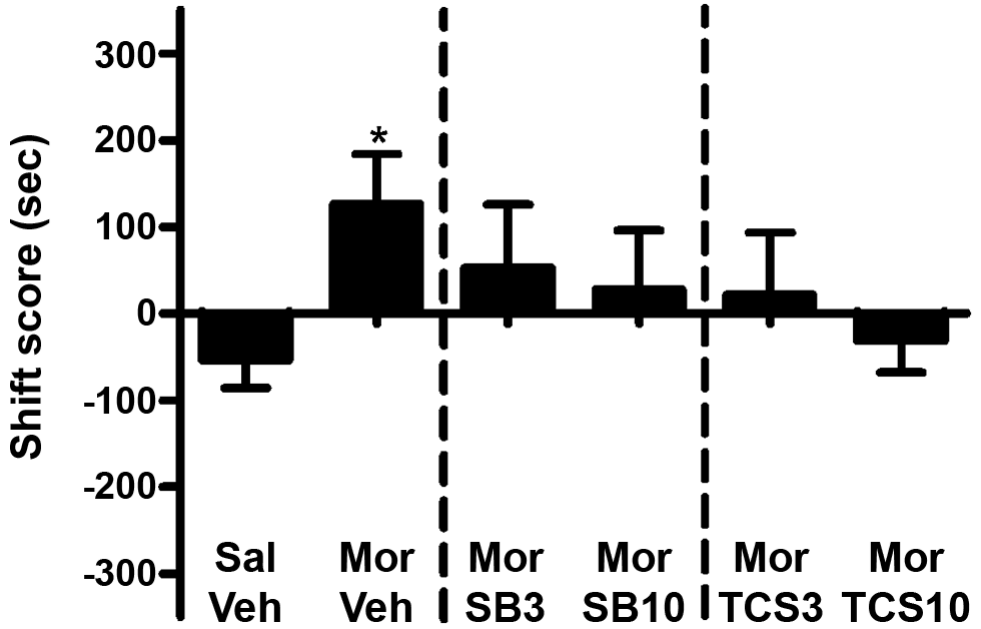
**Effects of microinjections of vehicle (Veh), SB334867 (SB) at 3.0 or 10.0 μg (SB3 and SB10), or TCSOX229 (TCS) at 3.0 or 10.0 μg (TCS3 and TCS10) in the NAcSh on morphine-induced reinstatement of morphine CPP.** *p < 0.05, the morphine group (Mor + Veh) compared to the control group (Sal + Veh). All data are expressed as mean ± SEM.

**Figure 6:**
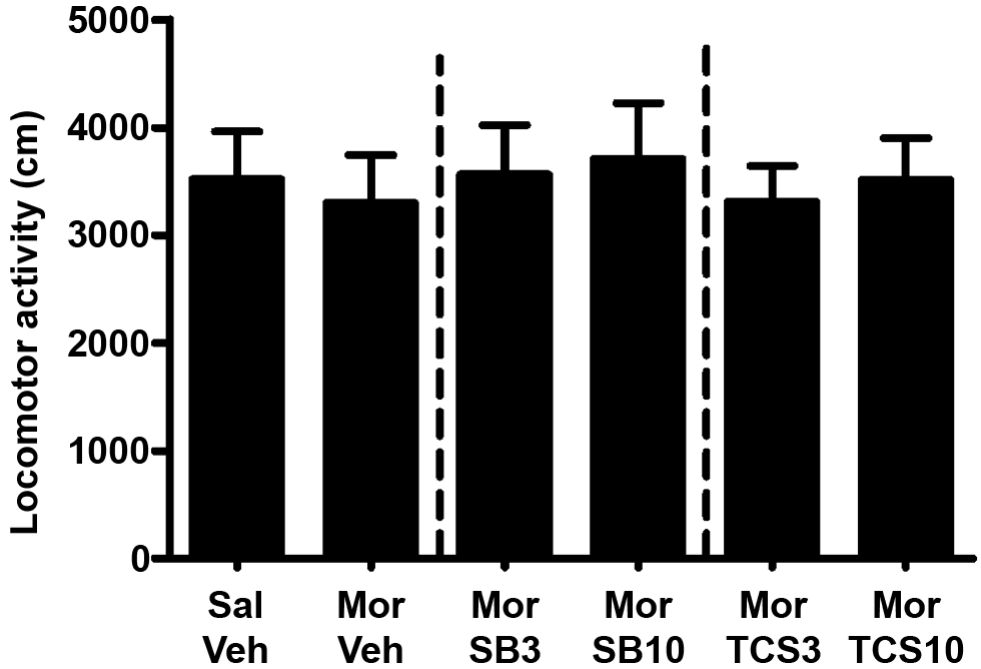
**Mean locomotor activity of all the groups in experiment 2.** All data are expressed as mean ± SEM.

As shown in Figures 5 and 6, independent samples *t* test showed a significant difference between the morphine priming group and the control group (*t*(20) = 2.554, *p* = 0.019), indicating the 3.0 mg/kg (i.p.) morphine injection remarkably reinstated the extinguished morphine CPP. To examine the effect of OX1R or OX2R antagonist on the morphine-induced reinstatement, the rats received two doses of SB334867 or TCSOX229 microinjection into the NAcSh. One-way ANOVA demonstrated that neither SB334867 (*F*(2,31) = 0.610, *p* = 0.550) nor TCSOX229 (*F*(2,31) = 2.210, *p* = 0.127) had any effect on the reinstatement of CPP or locomotor activity (*F*(2,31) = 0.199, *p* = 0.821; *F*(2,31) = 0.098, *p* = 0.907).

## Discussion

Recent studies have demonstrated an important role for the orexin system in relapse to drugs of abuse, especially to cocaine and alcohol, but little is known about the role of orexins in opioid relapse (Plaza-Zabala et al., 2012). In the present study, we found that footshock stress and morphine challenge significantly reinstated the extinguished morphine CPP. The footshock-induced reinstatement was attenuated by the blockade of OX1R or OX2R in the NAcSh, but neither of these two orexin receptor antagonists prevented morphine-induced reinstatement when injected in the NAcSh. These findings suggest that the NAcSh is a potential brain site for orexin to mediate the stress-induced opioid relapse.

Besides its role in positive emotions, the NAcSh has also been indicated in negative motivational states, such as fear, defensive behavior and especially stress (Reynolds and Berridge, 2001). Studies showed that many neurotransmitters (e.g., dopamine, glutamate, 5-hydroxytryptamine (5-HT) and γ-aminobutyric acid (GABA)) within the NAcSh mediated these negative motivations (Inoue et al., 1994; Reynolds and Berridge, 2001; Richard and Berridge, 2013). Our present study found that orexin signaling in the NAcSh, including OX1R and OX2R, is also a part of the neural mechanism of which the NAcSh participates in stress-related behaviors. Consistent with our results, a previous study showed that footshock stress was able to activate orexin neurons and increase the release of orexin (DiLeone et al., 2003).

Previous studies showed that systemic injection of the OX1R antagonist SB334867 attenuated footshock-induced reinstatement of cocaine seeking (Boutrel et al., 2005), and blockade of OX1R attenuate the reinstatement of alcohol seeking caused by yohimbine, an α2 noradrenergic antagonist that provokes a stress-like response (Richards et al., 2008). Whereas another study found that SB334867 had no inhibitory effect on footshock-induced reinstatement of nicotine seeking (Plaza-Zabala et al., 2010), it suggests that the role of OX1R in the stress-induced reinstatement is dependent on the types of abused drugs. The role of OX1R in the stress-induced effects of opioids (like morphine or heroin) reinstatement has not been reported previously.

The specific brain site through which OX1R works on the stress-induced reinstatement remains unknown. It was found that intra-VTA administration of SB334867 had no effect on the footshock-induced reinstatement of cocaine seeking (Wang et al., 2009). Here, we found that infusion of the antagonists of OX1R or OX2R into the NAcSh was able to prevent the footshock-induced morphine CPP reinstatement. It suggests that orexin signaling in the NAcSh is an important regulator to stress-induced morphine reinstatement. Therefore, the NAcSh is a crucial brain site through which OX1R modulates stress-induced reinstatement of morphine-seeking behavior.

A few studies examined the role of OX2R in stress and stress-induced relapse since the highly selective antagonist of the OX2R was not available until recent years, except a recent study showed that blockade of OX2R in the paraventricular nucleus of the thalamus (PVT) attenuated the anxiogenic states caused by a previous exposure to footshock stress (Li et al., 2010). Previous evidence showed that the NAcSh is an important brain site in footshock-induced reinstatement, indicated by that transient inactivation of the NAcSh blocked the footshock-induced reinstatement of cocaine-seeking behavior (Mcfarland et al., 2004). Although little is known about the receptor mechanisms underlying the NAcSh contribution to the footshock-induced drug relapse, a potential candidate is the dopamine system which is deeply involved in reward and motivation, and footshock increases extracellular dopamine in the NAcSh (Kalivas and Duffy, 1995; Pontieri et al., 1995; Kelley, 2004). Actually, antagonism of dopamine D1-like receptor in NAcSh but not in NAcC attenuated food-deprivation stress-induced reinstatement of heroin seeking behaviors (Tobin et al., 2013). As a main projection area of the orexin fibers, the NAcSh is likely to be one of the most important candidate brain areas where orexin may interact with dopamine to participate in footshock-induced drug relapse. Firstly, orexin fibers intermingled heavily with dopamine fibers within the caudal part of the NAcSh (Baldo et al., 2003); secondly, OXB predominantly excited the NAcSh neurons, and two-thirds of the neurons in this area increased their firing rate to two times when OXB and dopamine were simultaneously applied (Mori et al., 2011); thirdly, intra-NAcSh injection orexins increased dopamine concentration (Patyal et al., 2012); lastly, local injection of orexins in the NAcSh potentiated and OX1R antagonist attenuated dopamine receptor agonist-induced turning behaviors (Kotani et al., 2008). So, in the present study, it is likely that infusion of TCSOX229, a selective antagonist of OX2R, into the NAcSh may attenuate the ability of dopamine system to inhibit footshock-induced drug relapse.

Surprisingly, our findings showed that lower dose (3 μg) but not higher dose (10 μg) of OX1R or OX2R antagonist inhibited the footshock-induced reinstatement of CPP. Consistent with our results, a recent study also found that microinjection of lower dose (0.1 μg ), but not higher dose (1.0 μg ) of OX1R antagonist into the posterior part of the PVT decreased the stress-induced neuron activation and ACTH response (Heydendael et al., 2011). One potential interpretation for the negative effect of higher doses of orexin receptor antagonists may be the unspecific effect resulting from the relatively higher dose of the antagonists by binding to other receptors. For example, a recent study showed that higher dose of OX1R antagonist can act at 5HT_2C_ (serotonin 2C) receptors (Morairty et al., 2012). It has been established that blockade of 5HT_2C_ receptor is able to enhance cocaine reinstatement caused by stress and 5HT_2C_ agonist can attenuate drug seeking behaviors (Neisewander and Acosta, 2007; Fletcher et al., 2008; Higgins et al., 2012). In that case, higher dose of orexin antagonists may act in two ways, blockade of orexin receptor will attenuate the reinstatement, but on the other side, blockade of 5HT_2C_ receptor may enhance the reinstatement, therefore, it may be the possible reason that no effect of higher dose of orexin antagonists on the CPP reinstatement was observed in the present study. But further experiments are needed to examine whether 5HT_2C_ receptor is involved into the negative effect on the stress-induced morphine reinstatement by higher dose of orexin antagonists. It is also helpful for us to understand the pharmacological profile of orexin receptor antagonists with a third dose in the future studies.

Growing evidence has shown that there is functional difference between OX1R and OX2R, such as their different G protein coupling mechanisms, brain area distribution and ligand selectivity (Sakurai et al., 1998; Marcus et al., 2001; Zhu et al., 2003; Faedo et al., 2012). Signaling at OX1R is hypothesized to be primarily involved in reward-seeking behaviors based on the finding that cocaine-seeking was reinstated by local VTA microinjection of OXA but not OXB (Wang et al., 2009). OX1R signaling was also indicated to be involved in cue and context-induced reinstatement of cocaine seeking, while the antagonist of OX2R cannot block cue-induced cocaine seeking (Smith et al., 2009, 2010). Nevertheless, OX2R signaling is hypothesized to be related to arousal functions of the orexin system based on the finding that loss of the OX2R signaling is associated with symptoms of narcolepsy in animals (Lin et al., 1999; Willie et al., 2003; Akanmu and Honda, 2005; Dugovic et al., 2009). Since arousal is important for stress response, OX2R may participate in stress response and stress-induced reinstatement of drug-seeking behaviors. Here our study found that OX2R signaling was necessary for footshock-induced reinstatement of an extinguished morphine CPP. But we did not find a functional difference between OX1R and OX2R in footshock-induced reinstatement of opioid-seeking at the level of the NAcSh, since both of them worked on it.

Our results demonstrate that antagonism of neither of these two receptors in the NAcSh could block morphine-primed reinstatement. Consistent with our finding, a recent study found that OX1R signaling was unnecessary for cocaine-primed reinstatement of cocaine seeking (Aston-Jones et al., 2010). But little is known about the role of the OX2R signaling in drug-primed reinstatement. The neural mechanisms of the drug priming-induced reinstatement, which concerns many neurotransmitter systems, e.g., dopamine, glutamate, 5-HT and GABA, are very complicated and require further investigation (Shaham et al., 2003).

In conclusion, the NAcSh is probably an important part of the neural circuit where orexins exert a pivotal modulation of stress-induced reinstatement of opioid seeking behavior, but it may not be a critical brain site where orexin participates in drug-primed reinstatement of opioid seeking.

## Author contributions

Keke Qi conducted research, analyzed data and wrote paper; Chuguang Wei conducted research; Yonghui Li and Nan Sui designed research, wrote paper and had primary responsibility for final content.

## Conflict of interest statement

The authors declare that the research was conducted in the absence of any commercial or financial relationships that could be construed as a potential conflict of interest.
